# Prediction of the Limiting Flux and Its Correlation with the Reynolds Number during the Microfiltration of Skim Milk Using an Improved Model

**DOI:** 10.3390/foods9111621

**Published:** 2020-11-06

**Authors:** Carolina Astudillo-Castro, Andrés Cordova, Vinka Oyanedel-Craver, Carmen Soto-Maldonado, Pedro Valencia, Paola Henriquez, Rafael Jimenez-Flores

**Affiliations:** 1 Department of Food Engineering, Pontificia Universidad Católica de Valparaíso, Waddintgon 716, Valparaíso 2360100, Chile; paola.henriquez.c@mail.pucv.cl; 2 Civil and Environmental Engineering, College of Engineering, University of Rhode Island, Fascitelli Center for Advanced Engineering 317, 2 East Alumni Avenue, Kingston, RI 02881, USA; craver@uri.edu; 3 Regional Center for Studies in Healthy Food. Av. Universidad 330, Placilla, Sector Curauma, Valparaíso 2340000, Chile; carmensoto@creas.cl; 4 Department of Chemical and Environmental Engineering, Universidad Técnica Federico Santa Maria Avenida España 1680, Valparaíso 2340000, Chile; pedro.valencia@usm.cl; 5 Department of Food Science and Technology, The Ohio State University, Building 064, 2015 Fyffe Ct. Columbus, OH 43210, USA; jimenez-flores.1@osu.edu

**Keywords:** hydraulic diameter, limiting flux, Reynolds number, skim milk, microfiltration, ceramic membranes

## Abstract

Limiting flux (J_L_) determination is a critical issue for membrane processing. This work presents a modified exponential model for J_L_ calculation, based on a previously published version. Our research focused on skim milk microfiltrations. The processing variables studied were the crossflow velocity (CFV), membrane hydraulic diameter (d_h_), temperature, and concentration factor, totaling 62 experimental runs. Results showed that, by adding a new parameter called minimum transmembrane pressure, the modified model not only improved the fit of the experimental data compared to the former version (R^2^ > 97.00%), but also revealed the existence of a minimum transmembrane pressure required to obtain flux (J). This result is observed as a small shift to the right on J versus transmembrane pressure curves, and this shift increases with the flow velocity. This fact was reported in other investigations, but so far has gone uninvestigated. The J_L_ predicted values were correlated with the Reynolds number (Re) for each d_h_ tested. Results showed that for a same Re; J_L_ increased as d_h_ decreased; in a wide range of Re within the turbulent regime. Finally, from dimensionless correlations; a unique expression J_L_ = f (Re, d_h_) was obtained; predicting satisfactorily J_L_ (R^2^ = 84.11%) for the whole set of experiments

## 1. Introduction

The fractionation of milk compounds prior to their use is an interesting approach to obtain a maximum profits in the milk industry [1]. This approach is a good use of the fractions obtained from the milk and provides a base for the development of new products with functional properties [2]. For example, to concentrate casein micelles by microfiltration (MF) involves obtaining “native whey” in permeate. This process is gaining more attention from dairy producers because it reduces the large amounts of whey obtained during cheese making. Instead, the production of “native whey” leads to a more sustainable process because the whey has not suffered chemical modifications. Unlike the whey obtained from cheese making, native whey has no presence of added mineral salts, enzymes, and other process additives [3] that hamper the filtration process. In addition, soluble proteins such as β-lactoglobulin and α-lactoalbumin are in their native state. Therefore, native whey is considered the best starting point for obtaining proteins with intact functional properties [4].

Due to the “flux paradox”, experimental research on membrane technology is a must to predict proper operating conditions because the gel polarization model underpredicts the flux by 1 to 2 orders of magnitude in colloidal suspensions, hindering the selection of operating conditions that meet the needs of large-scale production [5]. This fact has led for the proposal of several models in order to better understand the mechanisms underlying a microfiltration process and to obtain more accuracy in flux prediction [5,6,7,8]. However, most of these models are only valid for some specific particle sizes and/or laminar flow ranges. The last does not reflect the reality since it is well known that microfiltration processes in the dairy industry are principally carried out in a turbulent regime [9]. Therefore, extreme care must be exercised to check the specifics of the case and compare these with the respective model assumptions [10].

To address the abovementioned difficulties, prediction of the limiting flux (J_L_) is a practical way to set up membrane operations. J_L_ corresponds to the maximum flux value obtained under some processing conditions that cannot be increased further by increasing the transmembrane pressure (ΔP_T_). A side effect of this condition is cake compaction, leading to fast flux decline and irreversible fouling [11]. On the other hand, the critical flux (Jc) theory proposed by Field et al. (1995) describes a condition where, despite reaching a lower initial flux value compared to the J_L_, irreversible fouling does not form on the membrane, and the operation performance is improved [12]. Based on this theory, Astudillo-Castro (2015) proposed an exponential model describing the nonlinear behavior of flux versus ΔP_T_ [13]. The model predicts the J_L_ and J_C_ along with the ΔP_T_ values at which they occur. It has been applied at different concentration factors and temperatures during the concentration of casein micelles by microfiltration [13] as well for the purification of prebiotic oligosaccharides by nanofiltration [14,15], providing an operational criterion that allows a stable flux. Regardless, it may be impractical to use this model at low ΔP_T_, as deviations between the experimental and predicted values have been observed. Normally, a shift in the J versus ΔP_T_ curve is expected when the osmotic pressure of the retained particles begins to become significant, as occurs in nanofiltration processes. However, milk proteins that are retained in microfiltration with 0.1 to 0.2 μm membranes would not generate such an effect because its osmotic pressures is negligible, and therefore, it is assumed that if J = 0, then ΔP_T_ = 0. However, it has been observed that the J versus ΔP_T_ curves for milk microfiltration do not necessarily start at that origin, instead there is a slight shift to the right. This phenomenon can be also observed directly from results presented by other authors during skim milk microfiltrations, as well as by extrapolation of the curves at low ΔP_T_ [9,16,17,18,19,20,21]. In practical terms this implies the existence of a minimal transmembrane pressure (ΔP_T_)_min_, even small, that must be applied to achieve permeate flux in microfiltrations. The best to our knowledge, this (ΔP_T_)_min_ has not been reported.

On the other hand, since flux also strongly depends on the hydrodynamic conditions, another practical approach for predicting the J_L_ is by its relationship with dimensionless numbers such as the Reynolds number (Re) [22]. Under laminar flow (Re < 2100) and constant CFV, a decrease in d_h_ increases the wall shear stress, which may result in an increased J_L_; however, there is not a simple relationship between the wall shear stress as a function of the CFV and d_h_ for turbulent flows such as those commonly used in skim milk microfiltration [20]. The unstable mixing of the fluid within the flow channel leads to differences in the way in which back-diffusion occurs [23]. When examining this subject further, the literature shows that previous researchers have found that the J_L_ is a linear function of the Re, but those works were performed by keeping the d_h_ constant [9,24,25]. Later, Hurt et al. (2015b) evaluated the effect of the d_h_ (3 mm and 4 mm) with respect the J_L_ obtained during skim milk microfiltration [20]. Results showed that the J_L_ was significantly lower for the 3 mm compared with that for the 4 mm d_h_ membranes, regardless of the protein concentration. Such differences were explained because different CFVs were used in each test (5.5 m/s and 7 m/s for the 3 and 4 mm d_h_ membranes, respectively) since the system was set for operating with a constant pressure drop, resulting in the CFV as a function of the d_h_. Despite the value of this work, J_L_ values were obtained by graphical representations instead of a model. The predictive J_L_ exponential model [13] proposed by Astudillo-Castro (2015) turns out to be practical, but in light of the aforementioned discussion, several research questions are raised: What drive the displacement of the J vs. ΔP_T_ curves resulting on the appearance of a ΔP_T,min_ > 0? How much the robustness of the J_L_ prediction improve by including ΔP_T,min_ (offset in the *x*-axis) in the model previously reported? Despite several works that have related J_L_ and Re by empirical equations, what is the relationship between J_L_ and Re? Are a function type J_L_ = f (Re) enough to describe the phenomena such as was previously reported?

The objectives of this research were to assess the modification of the J_L_ prediction model proposed by Astudillo-Castro (2015) by considering the displacement of the operational curves from the origin on skim milk microfiltrations performed at different processing conditions, that is, flow, temperature, concentration factor, and d_h_. Our second objective was to study if there is a single relationship between the J_L_ (obtained by the modified exponential model) and the Re regardless of the d_h_ of the membrane and to identify the hydrodynamic parameters that cause this eventual relationship. Finally, we aimed to deliver a single expression for predicting the J_L_ for the entire set of conditions studied.

## 2. Materials and Methods

### 2.1. Materials

**Figure 1:**
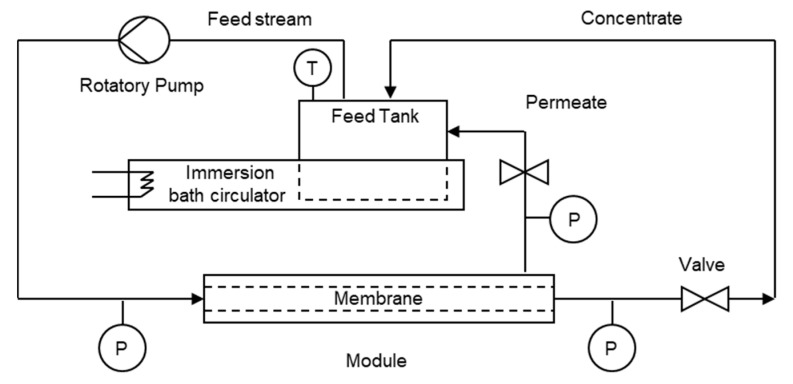
Full recirculation mode setup. P and T are the pressure and temperature gauges, respectively.

A Membralox T1-70 module (Pall) for ceramic membranes (0.14 μm, Tami) of 10 mm in external diameter and 25 cm in length was used. Three hydraulic diameters (d_h_) of 2, 3.6, and 6 mm were tested, and the filtration areas for these membranes were 0.01090 m^2^, 0.00940 m^2^, and 0.00147 m^2^, respectively. The membrane area differences are due the number of channels in each one, as detailed in Table 1. Figure 1 shows the experimental setup.

Three rotatory vane pumps (Fluid o-Tech) were used in order to obtain different flows inside the module. The experiments were carried out under constant flow conditions. Table 1 shows the information on the flow (Q) provided by the pumps and the CFV. Additionally, an analytical balance (Radwag, WTB 2000, Radom, Poland) connected to a computer recorded the measuring permeate mass, in order to compute flux (J) in terms of L/(m^2^∙h).

The membrane resistance was calculated from plotting (J) flux versus ΔP_T_ data collected from a tests using deionized water at 50 °C and fitted to the classic resistance model [26] in Equation (1):

(1) $$J=\frac{{\Delta P}_{T}}{\mu \cdot R_{M}}$$

where ΔP_T_ is the transmembrane pressure (Pa), *μ* is the water viscosity at 50 °C (Pa·s), and R_M_ is the membrane resistance (m^−1^). For the new membranes, the average membrane resistance values were 4.57∙10^11^ m^−1^, 9.48∙10^11^ m^−1^, and 5.43∙10^11^ m^−1^ for the d_h_ values of 2, 3.6, and 6 mm, respectively. Moreover, in Supplementary Materials (Table S1), the properties and Re for water are given.

Commercial low heat skimmed milk powder (Hormel Foods, MN, USA) was used for preparing all milk solutions with different total protein concentrations (1.5, 3, 4.5, and 9% *w/w*), thus representing different concentration factors (CF) of 0.5, 1.0, 1.5, and 3.0, respectively (for example, CF = 0.5 corresponds to diluted skim milk with half of the solids and casein concentration in regular skim milk). All of these milk solutions were reconstituted [13] with deionized water (<5 μS/cm). The average particle diameter for the reconstituted skim milk was determined using a laser diffraction (Mastersizer X, Malvern Instruments, 0.63 µm laser wavelength, MSX1, UK). Table 2 summarizes the physical properties of each milk solution. The density was measured as stated elsewhere, and the viscosity was determined on a DV - II + Pro Brookfield viscometer (Middleboro, MA, USA). For preventing the proliferation of microorganism’s, samples were treated with sodium azide (0.1%). All properties were measured in triplicate.

### 2.2. Experimental Runs

The equipment start-up and the flux versus transmembrane pressure curves were performed according to the methodology previously stated in detail [13]. The curves were performed with the skim milk solutions described above in the total recirculation mode to generate a pseudo-steady state. The permeate valve was manipulated manually, and the ΔP_T_ was gradually increased setting different values in the studied range (Table 1). For each experimental point, the flux versus time curves were drawn until a pseudo-stationary value (J∞) of flux was reached. Each step lasted 30 min, and for all of the experiments, J∞ was achieved within 30 min. Once this value was reached, average of J was calculated.

Each one of the membranes’ d_h_ of 2, 3.6, and 6 mm was treated as block of a Box-Bhenken experimental design with three central points considering the following factors: the flow rate (2.31, 5.31, and 7.85 L/min), the concentration factor (0.5, 1.0, and 1.5), and the temperature (40, 50, 60 °C), i.e., 18 runs per each membrane. To evaluate a wider range of Re values, some additional tests with 3.0 times the concentration factor in the mentioned flow rate and temperature ranges were also performed (Supplementary Materials Tables S2–S4). In this way, a total of 62 flux versus ΔP_T_ curves were performed to relate the J_L_ value with the Re.

After conducting each experiment, the membrane was rinsed with deionized water and cleaned using 1% (*w/v*) Ultrasil^®^ 11 at 60 °C and 1 bar in full recirculation mode during the first 30 min and in the concentration mode in the last 30 min [13]. Subsequently, two or three rinse cycles (45 min each) were performed, until no detergent residues were detected [27]. The effectiveness of the membrane cleaning procedure was then checked by measuring and comparing its hydraulic resistance to the initial membrane resistance, both determined using Equation (1).

### 2.3. Mathematical Modeling and J_L_ Prediction

Astudillo-Castro (2015) proposed that the variation in permeate flux, as a factor of the transmembrane pressure exerted, is proportional to the difference between the maximum permeate flux obtained experimentally (J_L_), and the flux observed at a certain ΔP_T_, according to the Equation (2):

(2) $$\frac{dJ}{d\Delta P_{T}}=\alpha \cdot \left(J_{L}-J\right),$$

Where *α* is a proportionality constant. The modified model considered that when the transmembrane pressure reaches the minimum value, there is no permeate flux, i.e., a minimum transmembrane pressure ((ΔP_T_)_Min_), where ΔP_T_ = (ΔP_T_)_Min_ → J = Then, by variable separation and integration of Equation (2), can be obtained Equation (3):

(3) $$\int_{0}^{J}\frac{dJ}{\left(J_{L}-J\right)}=\int_{{\left(\Delta P_{T}\right)}_{Min}}^{\Delta P_{T}}\alpha \cdot d\Delta P_{T}.$$

Integrating Equation (3) results in the following equation.

(4) $$\ln\left(\frac{J_{L}}{J_{L}-J}\right)=\alpha \cdot \left({\Delta P}_{T}-{\left({\Delta P}_{T}\right)}_{Min}\right).$$

Then, when clearing the flux value, Equation (5) is obtained

(5) $$J=J_{L}\left(1-\exp\left(-\alpha \cdot \left({\Delta P}_{T}-{\left({\Delta P}_{T}\right)}_{Min}\right)\right)\right).$$

If the Equation (5) is derivate and the resulting expression is evaluated at the intersection of axis of ΔP_T_ ((ΔP_T_)_Min_,0), then the slope of a tangent straight line from this point is obtained (J_L_·α). Therefore, the tangent straight line to the curve to ((ΔP_T_)_Min_, 0) is represented by the equation

(6) $$J=J_{L}\cdot \alpha \cdot \left({\Delta P}_{T}-{\left({\Delta P}_{T}\right)}_{Min}\right).$$

When ΔP_T_ → ∞, the limit for Equation (6) gives

(7) $$J=J_{L}.$$

To find the value for the critical transmembrane pressure ((ΔP_T_)_C)_), Equations (6) and (7) were intersected, with the aim to obtain the point ((ΔP_T_)_C_, J_C_).

(8) $$J_{L}=J_{L}\cdot \alpha \cdot \left({\left({\Delta P}_{T}\right)}_{C}-{\left({\Delta P}_{T}\right)}_{Min}\right)$$

Then, rearranging the terms, the value of α value can be determinate by

(9) $$\alpha =\frac{1}{{\left({\Delta P}_{T}\right)}_{C}-{\left({\Delta P}_{T}\right)}_{Min}}.$$

Finally, the modified exponential model is

(10) $$J=J_{L}\cdot \left(1-\exp\left(-\left(\frac{\Delta P_{T}-{\left(\Delta P_{T}\right)}_{Min}}{{\left(\Delta P_{T}\right)}_{C}-{\left(\Delta P_{T}\right)}_{Min}}\right)\right)\right).$$

Additionally, by evaluating Equation (10) at ΔP_T_ = (ΔP_T_)_C_, Equation (11) is obtained, and it directly relates the critical flux value with the limiting flux (J_L_).

(11) $$J\left[{\left(\Delta P_{T}\right)}_{C}\right]\;=\;J_{C}=0.632\cdot J_{L}.$$

Finally, for the calculation of the limiting transmembrane pressure (ΔP_T_)_L_, it was considered that this value was reached when the flux value was at least 95% of the limiting flux value, resulting in:

(12) $${\left(\Delta P_{T}\right)}_{L}\approx {\left(\Delta P_{T}\right)}_{Min}+3\cdot \left({\left(\Delta P_{T}\right)}_{C}-{\left(\Delta P_{T}\right)}_{Min}\right).$$

**Figure 2:**
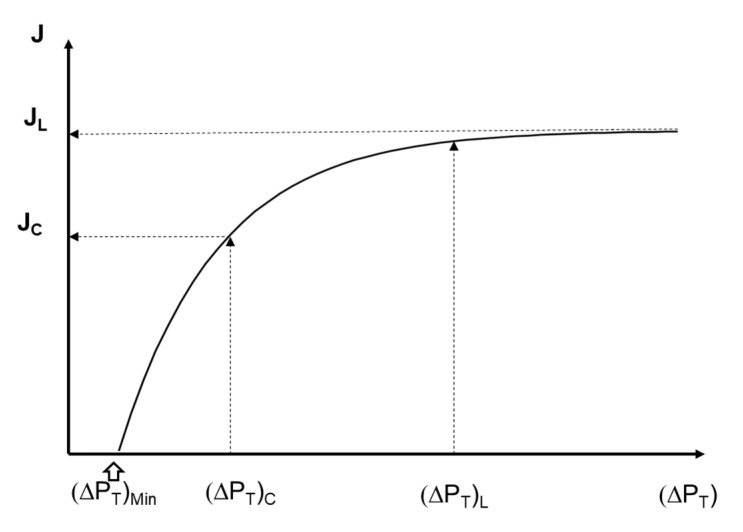
Generalized example of the graphical local of the parameter fitted by Equations (10)–(12).

Equation (10) has an asymptotic behavior towards J_L_ as ΔP_T_ is increased, and it is an easy way to obtain the critical flux J_C_, which is the point geometrically where a deviation from the linear relationship between the J and ΔP_T_ appears [12]. For clarity, Figure 2 summarizes the parameters calculated by this model in a J versus ΔP_T_ curve.

### 2.4. Chemical Analysis

The total protein contents (caseins and soluble proteins) in skim milk solutions were determined by the bicinchoninic acid method (BCA) using the Protein Research Reagents Kit (Pierce) [13]. The fraction of casein micelles was precipitated with acetic acid (1.2 M) until reaching their isoelectric point (pH = 4.6), and the supernatant containing the soluble proteins and lactose was subjected to precipitation with trichloroacetic acid [28,29]. The apparent rejection coefficients were calculated according to Suárez et al., (2006) [30].

### 2.5. Statistical Analyses

Data obtained from J versus ΔP_T_ curves were fitted in each case to Equation (10) using nonlinear squares minimum computations [31]. Results were expressed as the average flux taken when pseudo-steady state was reached. The parameters fitted by this procedure were J_L_, (ΔP_T_)_Min_, and (ΔP_T_)_C_. The goodness-of-fit of the experimental data to the model was evaluated using ANOVA regression analysis (*p* < 0.05), by plotting experimental values against predicted values in order to determine statistics related to the goodness-of-fit, specifically, the coefficient of correlation (R^2^) and the root mean square error (RMSE). Since experimental flux data were not significantly different compared to the J_L_ values predicted by Equation (10), the relationship between Re and J_L_ values was determined by using the predicted ones. Moreover, Kolmogorov-Smirnov tests were performed for checking if the residues fitted to a normal distribution, validating the models and detecting biases [31,32]. The same statistical validations were performed for the other expressions here presented.

## 3. Results

### 3.1. Flux Versus ΔP_T_ Curves: Effect of the Processing Conditions

**Figure 3:**
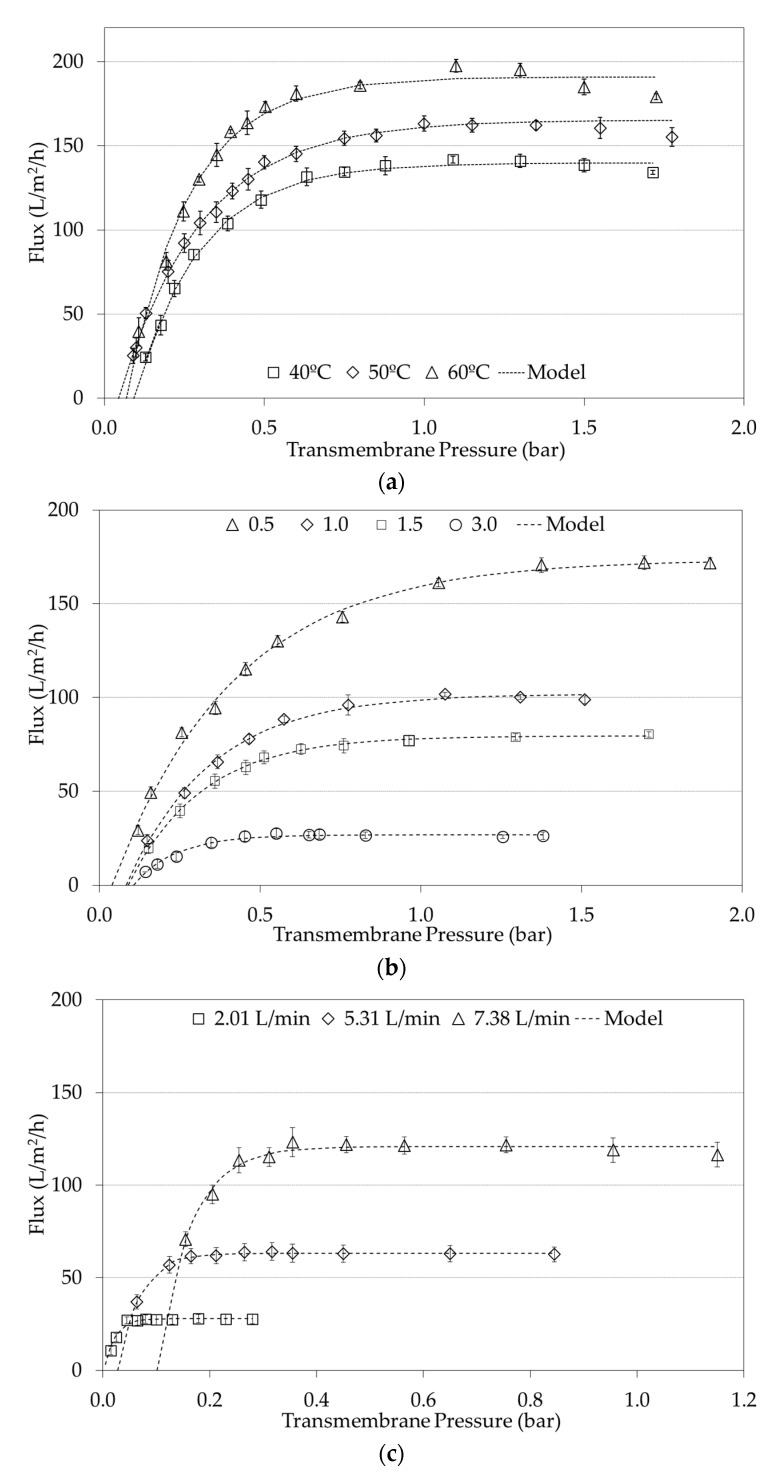
Effects of processing conditions on the flux versus transmembrane pressure. (**a**) Effect of Temperature. Experimental conditions: concentration factor of 0.5 at 7.38 L/min using the membrane with a d_h_ of 6 mm. (**b**) Effect of the Concentration Factor. Experimental conditions: flow of 7.38 L/min at 60 °C using the membrane with a d_h_ of 3.6 mm. (**c**) Effect of Flow. Experimental conditions: concentration factor of 1.5 at 60 °C using the membrane with a d_h_ of 2 mm_._

Since a total of 62 experiments were evaluated under a Box-Bhenken experimental scheme with complementary experiments, the Figures in this following section present the most illustrative results. Figure 3 considers the variables of temperature, concentration factor, and CFV, which had significant effects in the limiting flux [13,26]. The effect of temperature (40, 50, and 60 °C) is shown in Figure 3a for the membrane with a 6 mm using the concentration factor of 0.5 at 7.38 L/min. As expected, J_L_ increases significantly with temperature [13], a phenomenon observed regardless of the d_h_, FC, and flow values studied. Figure 3b shows the effect of the concentration factor (0.5, 1.0, 1.5, and 3.0) on the J vs. ΔP_T_ curves experiments conducted with a 3.6 mm d_h_ membrane, while keeping the temperature (60 °C) and flow (7.38 L/min) constant. These results clearly show how J_L_ decreases as the concentration factor increases, a situation well known to be involved in the membrane process due to an increased viscosity in the colloidal dispersion [13,33,34]. Finally, Figure 3c shows the effect of the flow (2.01, 5.31, and 7.38 L/min) on the J vs. ΔP_T_ curves with the membrane with a 2 mm d_h_ (temperature fixed at 60 °C and a concentration factor of 1.5). As expected, the increase in temperature and flow has a positive effect on the J_L_, but the concentration factor has a negative effect on this parameter [26,35]. Therefore, the J_L_ can be modified by manipulating the hydrodynamic conditions, such as flow or CFV, and the system physico−chemical properties, i.e., concentration factor and temperature [13]. It is worth noting the fact that all curves are offset from the origin to some extent, regardless of the process conditions. However, this effect becomes even more noticeable as the flow increases by shifting the curves to the right. In terms of the modified model, this can be reflected in a higher (ΔP_T_)_Min_. For example, in Figure 3c the (ΔP_T_)_Min_ at 2.01 L/min was 0.003 bar, but at 7.38 L/min it was 0.102 bar. The aforementioned values are also reported as fitted model parameters in Table 3. Besides, the osmotic pressure of casein micelles in milk is in the order of 1000 Pa [36,37], the existence of (ΔP_T_)_Min_ would be consequence of an interaction among osmotic pressure and the CFV applied. In practical terms, this finding implies the existence of a minimal transmembrane pressure (ΔP_T_)_min_ that must be applied to achieve permeate flux in microfiltrations. Despite this relatively small value, ((ΔP_T_)_min_) is important because the ΔP_T_ used during the operation are also small; therefore, (ΔP_T_)_min_ becomes numerically significant, as can be observed in Table 4 (see Section 3.3) when is compared with (ΔP_T_)_C_.

The above agrees with the well-known effect of increased flow resulting in increased CFV, the creation of turbulence, and changes in the hydrodynamic conditions [38,39], with the consequence of reducing membrane fouling [40]. An increased turbulence in turn increases the wall shear stress, increasing the back transport of particles to the bulk. Therefore, a minimum transmembrane pressure might be required, so that the flux of particles toward the membrane is greater than the back transport from the membrane, allowing the flux obtention. In summary, the higher the feed flow was, the greater the (ΔP_T_)_Min_ value required to obtain permeate flux. This finding can be observed in all the curves of Figure 3, confirming the need to modify the model previously published by Astudillo-Castro (2015) in order to improve the fitting of data and the accuracy in the predicted J_L_.

### 3.2. Effect of the Processing Conditions on the Protein Stability and Rejection

Owing to the stability of the proteins under the rheological conditions assayed, it was found that these conditions would not be sufficient to produce significant alterations in the whey proteins, nor would there be an effect due to the passage of milk into the pumps. For example, under high CFV and temperature levels (e.g., 4.64 m/s and 60 °C), the 7.7% of the whey proteins were denatured after 180 min of operation, while at 40 °C, no significant changes (*p* < 0.05) were observed (Supplementary Materials Figure S1). Therefore, a low whey protein denaturation effect was due to the increase in temperature instead of the CFV.

On the other hand, high casein micelle retention and low retention for soluble proteins are expected during skim milk microfiltration with membranes of 0.14 μm. In all experiments, the casein micelle apparent rejection coefficient was higher than 99.90% regardless of the experimental set up, probably caused by the average particle diameter of 0.24 µm for the reconstituted skim milk. Besides, the lowest soluble protein rejection was achieved at 60 °C at low ΔP_T_. For example, at ΔP_T_ lower than 0.5 bar and 60 °C, the apparent retention coefficient was in the range of 20 to 40%, which was expected for this kind of microfiltration [25,41,42].

### 3.3. Modified Exponential Model for J_L_ Prediction

**Figure 4:**
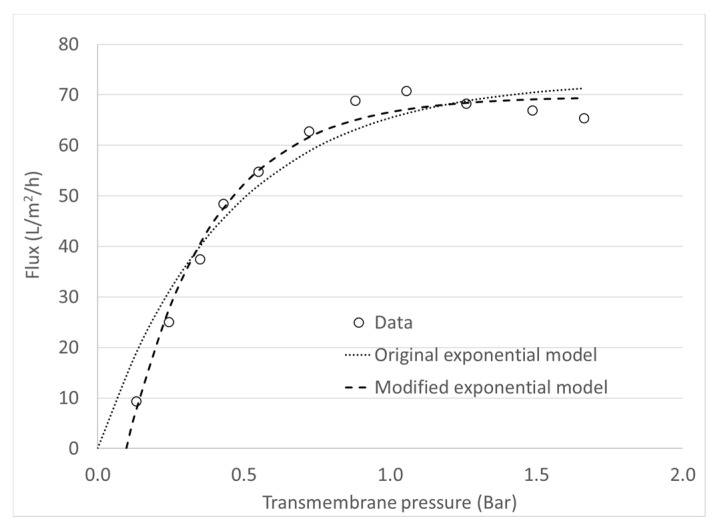
Comparison of data fitting to the original exponential model and the modified exponential model. Experimental conditions: Concentration factor of 1.0 with flow of 7.38 L/min at 40 °C using the membrane with a d_h_ of 3.6 mm.

Table 3 shows an example of the fitted parameters for the modified exponential model (Equation (10)) for 13 of the 62 performed, according to the curves shown in Figure 3 and Figure 4. All curves were fitted to the model with R^2^ > 97%. It is worth noting that for a good fit to Equation (10), the points of the J versus ΔP_T_ curve must be evenly distributed below and above the J_L_ value as shown in Figure 3. That means that the ΔP_T_ values must be increased to ensure completely encompassing the area limited by mass transfer. Additionally, since the model has 3 parameters to be adjusted, it is desirable that each curve has at least 6 experimental points (which is the most observable case in literature) improving the robustness of the prediction model [31]. Hence, the addition of the parameter (ΔP_T_)_Min_ to the original exponential model improved data fit for the modified one, without significantly affecting the model parsimony.

Figure 4 shows an example when average flux data is plotted with the original exponential model and the modified exponential model. It is evident how incorporating this new parameter not only provides a physical interpretation of a phenomenon that was previously disregarded, but also reduces the global distance that exists between the experimental points and the values predicted by the model. In statistical terms, this distance is measured by the Root Mean Square Error (RMSE). In Figure 4, the RMSE of the original model was 4.89, while in the modified model it was 2. The reduction of the RMSE was observed in all the experimental runs, regardless of the processing conditions when using the modified equation (not shown data). This improvement therefore translates into a lower error in the prediction of the value of J_L_, resulting in a better accuracy when building a relationship between this parameter and the Re. In addition, for all of the curves of J vs. ΔP_T_, the ANOVA regressions yielded significant fitting of the model to the experimental data (*p* < 0.05), and no bias was detected because according to the Kolmogorov-Smirnov tests the residues fitted to a normal distribution (*p* < 0.005).

### 3.4. Effect of the d_h_ on the J_L_

**Figure 5:**
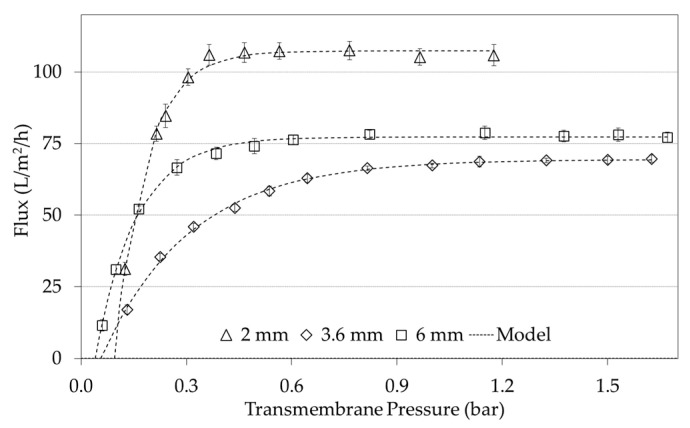
Flow of 7.38 L/min. Experimental conditions: concentration factor 1.5 at 50 °C.

Figure 5 shows an example of the effect of the d_h_ on J versus ΔP_T_ curves at the highest flow (7.38 L/min), at CF = 1.5 and 50 °C. It is interesting to note the order of greatest to smallest J_L_ values that the curves had for all cases—i.e., keeping constant the process variables except d_h_—was the same behavior: J_L 2 mm_ > J_L 6 mm_ > J_L 3.6 mm_, as can be seen in Figure 5.

This result could occur because, for the same flow, the CFV changed as result of the d_h_. According to Table 1, CFV_2mm_ > CFV_6mm_ > CFV_3.6mm_, which is same order of the J_L_ obtained for each case. However, the CFV_6 mm_ was slightly higher (8.0%) than CFV_3.6 mm_. Then, the fact of J_L 6mm_>J_L 3.6mm_ is a result of $\tau_{W}$_6mm_>$\tau_{W}$_3.6mm_. Indeed, *τ*_w_ is defined in Equation (13):

(13) $$\tau_{w}=\frac{\Delta P\cdot d_{h}}{4\cdot L},$$

where Δ*P* is the drop pressure, d_h_ is the hydraulic diameter, and *L* is the membrane length [43]. Hence, the higher *d_h_* higher *τ*_w_. Besides Δ*P* is a quadratic function of CFV, therefore, a higher CFV implies higher Δ*P*. Le Berre and Daufin (1996) showed that the effect of an increase in the shear stress generates a qualitative and quantitative effect during microfiltration since a decrease in the porosity and thickness of the layer deposited on the membrane is achieved, thus removing the casein micelles back into the bulk suspension, while soluble proteins break through the membrane [44]. In this way, the concentration in the boundary layer cannot remain constant, leading to a significant improvement in the flux [45].

### 3.5. Effect of the d_h_ on the Relationship between J_L_ and Re

There are a few investigations that relate the limiting flux with Reynolds number. Table 4 lists most of correlations that have been reported between the Reynolds number and the limit flux for skim milk microfiltration. The correlations submitted by Samuelsson et al. (1997a, 1997b) show a linear relationship between Re and J_L_. Krstić et al.’s work (2002) shows several expressions where J is proportional to Re up to c (J α Re^c^), where c < 1 for all membranes analyzed with or without turbulence promoters [24,25,46]. However, Krstić et al. (2002) did not report the limiting flux [46], but they reported the permeate flux measured under constant transmembrane pressure. However, according to the J versus ΔP_T_ curve inspection, it can be observed that the transmembrane pressure used by them would be in a zone where the limiting flux was already achieved; therefore, it was possible to compare. Later, Baruah et al. (2003) found a linear relationship between J_L_ and Re, but they did not report any expression for relating both parameters [6]. On the other hand, Gésan-Guiziou et al. (1999) observed that J_C_ increased differently with Re, when different d_h_ are used [43]. Most recently, Hurt et al. (2015) reported a linear relationship using ceramic membrane with graded permeability, but they were not able to obtain a single correlation for their results using two membranes with d_h_ values of 3 and 4 mm [20]. They introduced a modified Reynolds number by substituting the hydraulic diameter for the membrane length, obtaining a correlation of R^2^ = 92.09% (*n* = 18).

It is worth mentioning that in some works shown in Table 4, the temperature and/or concentration factor were kept constant. In these cases, the Re was modified only by the effect of the CFV. For example, Krstić et al.’s work (2002) was carried out for a concentration factor of 1.0 during milk microfiltration in a full recirculation mode [46]. The exception is work of Krstić et al., (2004) where different concentration factors (1 to 2) [47] were used; however, no mathematical relationships were reported.

**Figure 6:**
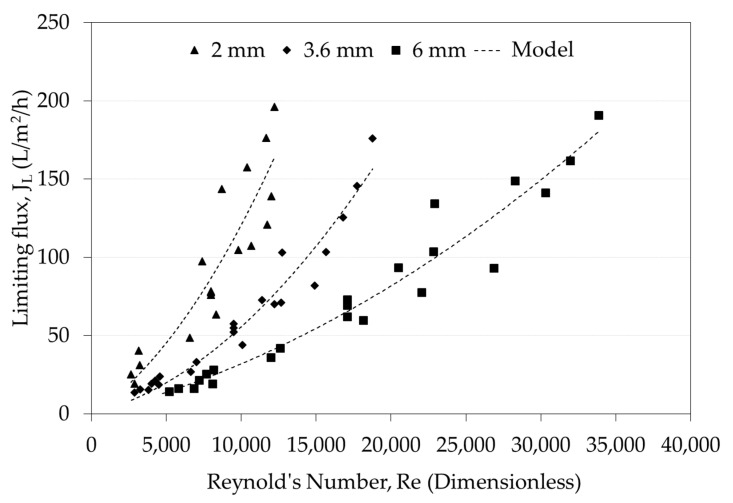
Limiting flux versus Reynolds number curves during skim milk microfiltration for three hydraulic diameters (2, 3.6, and 6 mm). Model type: J_L_ = (aRe + b) Re.

Figure 6 shows the J_L_ versus Re for three hydraulic diameters, i.e., 2, 3.6, and 6 mm d_h_ membranes, considering the total 62 experimental runs. It can be observed that the three curves have the same shape, but for the same turbulence state (i.e., the same Reynolds number), the J_L_ values are inversely proportional to the d_h_, that is J_L 2mm_ > J_L 3.6mm_ > J_L 6mm_. This result could occur because smaller channels increase the back transport of substances towards the bulk solution by reducing the concentration of particles on the membrane surface and have the effect of reducing the polarization concentration, which consequently increases the flux [48].

Despite that, for each hydraulic diameter, a fine correlation could be found for the J_L_ versus Re, which is a dimensionless number that does not explain the difference in the J_L_ among the three d_h_ tested. Similar behavior was found for 3 and 4 mm d_h_ during milk microfiltration [20]. The higher dispersion of data was found for the 2 mm membrane, this could be explained due to the constriction from the flow channel (10 mm) to the membrane channel (2 mm) is the greatest.

Table 5 shows the equations for the correlations obtained for each d_h_ membrane by considering linear models. A generalized linear model for this case can be expressed as:

(14) $$J_{L}=a\cdot Re$$

Moreover, a quadratic model was considered based on the shape of the curves shown in Figure 6:

(15) $$J_{L}=\left(a\cdot Re+b\right)\cdot Re$$

In both types of correlations J_L_ has the same units (L/m^2^/h). It can also be observed that relationship models of Re to J_L_ exhibited satisfactory fitting since all R^2^ were higher than 77.43%. Additionally, ANOVA regression routines showed that all data were significantly represented by these equations (*p* > 0.05), while the Kolmogorov-Smirnov test performed on the residues showed no bias (data not shown), thus demonstrating the validation of these correlations. In the linear models, a greater increase in the slope values (*a*) is observed as the d_h_ decreases. This increase means that for the same Re, there is a greater effect on the J_L_ as the hydraulic channel becomes thinner. On the other hand, the quadratic models had better fits compared to the linear models, but included an additional parameter, thus reducing in one degree of freedom. In this case, the *a* values were inversely proportional to the d_h_, while the parameter *b* also tends to decrease to some extent as the hydraulic channel widens. It is worth mentioning that all of these correlation models are valid under transition to turbulent regime since Re > 2300.

For the large Re range studied in this work, it is clear that quadratic behavior fits better to experimental data, compared with the previous works showed in Table 4. Hence, the phenomenon obeys to a potential law (*n* > 1).

Based on the above, and with the aim of obtaining one single expression, the dimensionless correlation between Sherwood (Sh), Schmidt (Sc), and Re [49] was considered:

(16) $$Sh=\frac{k\cdot d_{h}}{D}=a\cdot Re^{b}\cdot Sc^{c}.$$

Then, by replacing the definition of *Sh* = *k∙d_h_/D*, where *D* is the diffusion coefficient, and clearing the mass transfer coefficient (*k*), Equation (17) is obtained.

(17) $$k=a\cdot \frac{D}{d_{h}}\cdot Re^{b}\cdot Sc^{c}.$$

Under steady-state conditions, the convective solute flow to the membrane surface will be balanced by the solute flux through the membrane plus the diffusive flow from the membrane surface to the bulk [49], giving the following expression for boundary layer:

(18) $$\left(\frac{C_{G}-C_{P}}{C_{B}-C_{P}}\right)=Exp\left(\frac{J}{k}\right),$$

where *C_G_*, *C_B_*, and *C_P_* are the solute concentration in the gel layer, bulk, and permeate, respectively. By clearing k, Equation (19) is obtained.

(19) $$k=J\cdot Ln\left(\frac{C_{B}-C_{P}}{C_{G}-C_{P}}\right).$$

By replacing Equation (19) in Equation (17) and clearing *J*:

(20) $$J=a\cdot Ln\left(\frac{C_{B}-C_{P}}{C_{M}-C_{P}}\right)\cdot \frac{D}{d_{h}}\cdot Re^{b}\cdot Sc^{c}.$$

Moreover, in steady state *J* = *J_L_* and considering all the other variables, except *Re* and *d_h_*, absorbed by a new constant value called $a^{\prime }$, the Equation (21) is obtained.

(21) $$J_{L}=a^{\prime }\cdot \frac{Re^{b}}{d_{h}}.$$

By correlation of the data in this current investigation, the Equation (22) is obtained for *J_L_* in L/m^2^/h and *d_h_* in m.

(22) $$J_{L}=3.29\cdot 10^{-6}\cdot \frac{Re^{1.21}}{d_{h}}.$$

**Figure 7:**
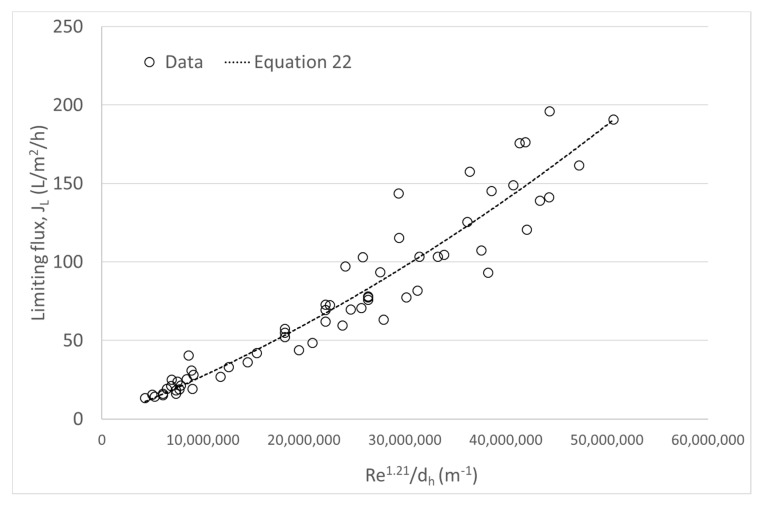
*J_L_* versus Re^b^/*d_h_* curve during skim milk microfiltration using data from three hydraulic diameters: 2, 3.6, and 6 mm (*n* = 62), where b was fixed at 1.21.

Equation (22) shows the correlation of *J_L_* = f (Re,*d_h_*) for the whole set of experimental conditions (*n* = 62), considering simultaneously the 3 *d_h_* values studied. The correlation robustly explained the variability of the phenomenon (R^2^ = 88.97% and RSME = 16.93). For graphical purposes, *J_L_* vs. Re^b^/*d_h_* was depicted, obtaining Figure 7.

Finally, a single expression for predicting J_L_ as function of the Re and d_h_ has been found. Therefore, this kind of expression which comes from a dimensionless number is more appropriate for describe this phenomenon.

## 4. Conclusions

A previously reported exponential model for J_L_ determination in skim milk microfiltration was modified. The inclusion of a new parameter, (ΔP_T_)_Min_, reflected the existence of minimum transmembrane pressure required to obtain flux. This phenomenon has been generally obviated, but literature suggests its appearance in skim milk microfiltrations. From the 62 processing conditions assayed, results showed that the higher the feed flow, the greater the value of (ΔP_T_)_Min_ as result of rightward shift of the J versus ΔP_T_ curves. This fact can be explained because an increased turbulence increases the wall shear stress, thus increasing the back transport of particles to the bulk. In this way, the modified exponential model not only provides a parameter with physical meaning, but also substantially improved the fit of the data of the operational curves compared to the former model version. The above involves obtaining J_L_ predictions with better accuracy.

On the other hand, a nonlinear empiric relationship between J_L_ and the Re was obtained for each d_h_. Results showed that for a same Re, J_L_ increased as d_h_ decreased, in a wide range of Re within the turbulent regime (2653 < Re < 33846). Even though these correlations had an adequate degree of adjustment (R^2^ ≥ 81.36%), they were only valid for one d_h_. From dimensionless correlations a unique expression J_L_ = f(Re,d_h_) was obtained that satisfactorily predicted the J_L_ (R^2^ = 84.11%). The practical implications of this finding are useful when selecting the membranes for a skimmed milk MF process; for the same level of turbulence, membranes with a lower d_h_ will produce higher fluxes with a better packing capacity. However, the use of membranes with lower d_h_ creates a higher pressure drop at the same flow rate, which involves higher cost for electricity for pumping and cooling. All these consequences must be evaluated simultaneously to select a proper d_h_, resulting in a truly more sustainable process.

## Figures and Tables

**Table 1:** Flow and crossflow velocity (CFV) for each pump and hydraulic diameter.

Pump	ΔP_T_ Working Range (bar)	Average Flow, Q (L/min)	Average Cross Flow CFV (m/s)		
			d_h_ = 2 mm (7 Channels)	d_h_ = 3.6 mm (3 Channels)	d_h_ = 6 mm (1 Channel)
0711	0.02–0.7	2.01 *	1.52	1.10	1.18
2511	0.1–1.5	5.31 *	4.02	2.90	3.13
411	0.1–1.8	7.85 *	5.95	4.28	4.63

(*) Values experimentally measured and previously reported by Astudillo-Castro (2015).

**Table 2:** Physico-chemical properties of the tested skim milk solutions.

Total Protein (% *w/w*)	1.5	3	4.5	9	Temperature (°C)
Concentration Factor	0.5	1	1.5	3	
Density (kg/m^3^)	994.8 ± 3.5	1011.8 ± 6.1	1023.4 ± 1.5	1075.7 ± 4.4	40
	990.7 ± 5.7	1007.7 ± 3.7	1021.8 ± 3.1		50
	986.0 ± 6.3	1002.9 ± 2.8	1015.4 ± 0.7	1073.2 ± 3.2	60
Viscosity (cP)	0.912 ± 0.010	1.231 ± 0.014	1.525 ± 0.009	3.904 ± 0.022	40
	0.861 ± 0.010	1.108 ± 0.013	1.288 ± 0.008		50
	0.809 ± 0.013	0.986 ± 0.009	1.051 ± 0.011	2.490 ± 0.018	60

**Table 3:** Model prediction and parameters determination (average values).

T (°C)	CF	Q (L/min)	d_h_ (mm)	J_L_ (L/m^2^/h)	(ΔP_T_)_C_ (bar)	(ΔP_T_)_Min_(bar)	R^2^	RMSE	Figure
40	0.5	7.38	6	139.7	0.299	0.092	99.95	2.44	3a
50	0.5	7.38	6	165.3	0.303	0.045	99.51	1.83	3a
60	0.5	7.38	6	190.8	0.266	0.068	98.61	5.3	3a
60	0.5	7.38	3.6	173.7	0.383	0.039	99.95	3.04	3b
60	1.0	7.38	3.6	101.8	0.264	0.083	99.96	1.61	3b
60	1.5	7.38	3.6	79.5	0.230	0.089	99.87	0.71	3b
60	3.0	7.38	3.6	26.7	0.131	0.106	97.52	1.01	3b
60	1.5	2.01	2	27.90	0.022	0.003	98.67	0.83	3c
60	1.5	5.31	2	63.31	0.069	0.028	99.99	0.54	3c
60	1.5	7.38	2	120.8	0.163	0.102	97.34	2.70	3c
50	1.5	7.38	2	107.4	0.184	0.095	99.47	1.66	5
50	1.5	7.38	3.6	69.5	0.309	0.056	99.70	1.00	5
50	1.5	7.38	6	77.4	0.157	0.040	99.77	1.01	5

**Table 4:** Correlations between the limiting flux and the Reynolds number reported in the literature.

Membrane and Experimental Conditions	Equation			Reference
Ceramic 0.14 μm; d_h_ = 6 mm; L = 138 mm, Skim milk MF. Data (*n* = 8) was adjusted to an empirical relation.	J_L_ (m/s) = 6.94·10^−10^·Re			[24]
Ceramic 0.14 μm; d_h_ = 6 mm; v = 1.5–8 m/s; T = 15 and 55 °C, Skim milk MF. (Length no reported, but the filtration area was 26 cm^2^, implying L = 138 mm). Data (*n* = 8) was adjusted to an empirical relation.	J_L_ (L/(m^2^·h)) = 0.0025 Re			[25]
Ceramics (0.05, 0.1 and 0.2 µm); T = 50 °C, Skim milk MF; v ≥ 0.45 m/s, with and without turbulence promoters; ΔP_T_ = 65 kPa. L = 250 mm and d_h_ = 6.8 mm. Data was adjusted to an empirical relation.	J α Re^0.15^	Re < 2700	Without turbulence promoter	[46]
	J α Re^0.80^	Re > 2700		
	J α Re^0.85^	Re > 2000	With turbulence promoter	
Ceramics 0.1 µm (3 mm d_h_ ceramic graded permeability and 4 mm d_h_); T = 50 °C. Milk with different total protein concentrations (8–9–10%). L = 1.02 m. Data was adjusted to an empirical relation.	J_L_ (kg/m^2^/h) = 0.00764·Re Only for the 4 mm d_h_ membrane using *n* = 9.			[20]
	J_L_ (kg/m^2^/h) = 3.07·10^−5^ Re_Length based_ where Re_Length based_ is a modified Re as follows: ReLegth based =ρ·CFV·Lμ where *L* is the membrane length, which is 1.02 m for both membranes. In this case *n* = 18.			

**Table 5:** Correlations between the limiting flux and the Reynolds number for skim milk MF.

Hydraulic Diameter	Range	Equation	Determination Coefficient (R^2^)	RSME
d_h_ = 2 mm	2653 < Re < 12234	J_L_ = (5.81·10^−7^·Re + 6.20·10^−3^) Re	81.73%	19.72
		J_L_ = 0.01204·Re	77.46%	21.91
d_h_ = 3.6 mm	2658 < Re < 18773	J_L_ = (3.17·10^−7^·Re + 2.38·10^−3^)·Re	95.23%	9.77
		J_L_ = 0.00681·Re	86.10%	16.69
d_h_ = 6 mm	4752 < Re < 33846	J_L_ = (8.95·10^−8^·Re + 2.23·10^−3^)·Re	95.33%	11.10
		J_L_ = 0.00451·Re	89.46%	16.68

## Supplementary Materials

The following are available online at https://www.mdpi.com/2304-8158/9/11/1621/s1, Figure S1: Evolution of the percentage of proteins respect their original concentration under extreme shear stress conditions: 231 Pa at 40 °C, and shear stress of 214 Pa at 60 °C, Table S1: Properties and Reynolds number for wáter, Table S2: Experimental runs and results for JL according to Box Behnken design for the 2 mm dh membrane, Table S3: Experimental runs and results for JL according to Box Behnken design for the 3.6 mm dh membrane and additional runs, Table S4: Experimental runs and results for JL according to Box Behnken design for the 6 mm dh membrane and additional runs.
