# Supplementary material for: Prediction of the Limiting Flux and Its Correlation with the Reynolds Number during the Microfiltration of Skim Milk Using an Improved Model
Source: Foods. 2020 Nov 6;9(11):1621. doi: 10.3390/foods9111621 (PMC7695011; doi:10.3390/foods9111621)
Supplement: Supplementary file 1 [file foods-09-01621-s001.pdf]

foods

Supplementary Material

Table S1: Properties and Reynolds number for water

Temperature (°C)	Viscosity (cp)	Density (kg/m³)	d_h=2 mm			d_h=3.6 mm			d_h=6 mm		
			Flow (L/min)								
			**2.01**	**5.31**	**7.85**	**2.01**	**5.31**	**7.85**	**2.01**	**5.31**	**7.85**
**40**	0.78	992.2	3876	10239	15136	5024	13272	19621	9043	23890	35317
**50**	0.7	988.0	4300	11360	16795	5574	14727	21771	10034	26508	39188
**60**	0.62	983.2	4831	12764	18869	6263	16545	24460	11273	29782	44028

Table S2: Experimental runs and results for J_L according to Box Behnken design for the 2 mm d_h membrane.

Block	CF	Flow (L/min)	T (ºC)	J_L (L/m^2/h)
Box Behnken for d_h= 2 mm	0.5	2.01	50	40.5
	0.5	5.38	40	97.4
	0.5	5.38	60	143.7
	0.5	7.38	40	157.5
	0.5	7.38	50	176.4
	0.5	7.38	60	196.1
	1.0	2.01	40	25.2
	1.0	2.01	60	31.0
	1.0	5.38	50	77.6
	1.0	5.38	50	78.2
	1.0	5.38	50	76.0
	1.0	7.38	40	104.6
	1.0	7.38	60	139.0
	1.5	2.01	50	19.1
	1.5	5.38	40	48.5
	1.5	5.38	60	63.3
	1.5	7.38	50	107.4
	1.5	7.38	60	120.8

Table S3: Experimental runs and results for J_L according to Box Behnken design for the 3.6 mm d_h membrane and additional runs

Block	CF	Flow (L/min)	T (ºC)	J_L (L/m²/h)
Box Behnken for d_h= 3.6 mm	0.5	2.01	50	23.8
	0.5	5.38	40	72.7
	0.5	5.38	60	103.1
	0.5	7.38	40	125.5
	0.5	7.38	50	145.4
	0.5	7.38	60	175.9
	1.0	2.01	40	15.6
	1.0	2.01	60	19.3
	1.0	5.38	50	54.9
	1.0	5.38	50	57.6
	1.0	5.38	50	52.1
	1.0	7.38	40	70.9
	1.0	7.38	60	103.3
	1.5	2.01	60	15.3
	1.5	5.38	40	33.0
	1.5	5.38	60	44.0
	1.5	7.38	50	69.9
	1.5	7.38	60	81.8
Adicional runs	3.0	5.38	40	13.6
	3.0	5.38	60	18.5
	3.0	7.38	40	21.3
	3.0	7.38	60	26.9

Table S4: Experimental runs and results for J_L according to Box Behnken design for the 6 mm d_h membrane and additional runs

Block	CF	Flow (L/min)	T (ºC)	J_L (L/m²/h)
Box Behnken for d_h= 6 mm	0.5	2.01	50	28.2
	0.5	5.38	40	93.5
	0.5	5.38	60	134.5
	0.5	7.38	40	141.2
	0.5	7.38	50	161.7
	0.5	7.38	60	190.8
	1.0	2.01	40	16.3
	1.0	2.01	60	21.5
	1.0	5.38	50	73.0
	1.0	5.38	50	62.1
	1.0	5.38	50	69.4
	1.0	7.38	40	103.5
	1.0	7.38	60	148.9
	1.5	2.01	60	16.1
	1.5	5.38	40	42.0
	1.5	5.38	60	59.7
	1.5	7.38	50	77.5
	1.5	7.38	60	93.3
Adicional runs	3.0	5.38	40	14.4
	3.0	5.38	60	19.3
	3.0	7.38	40	25.5
	3.0	7.38	60	36.1

Protein Stability

Milk has two main fractions of proteins: micelle caseins and soluble proteins. The second fraction is highly denaturized by temperature. According to Pelegrine & Gasparetto (2005), the solubility of soluble proteins in milk is an indicator of their native state [1], therefore the protein that remains soluble with no precipitation at pH 4.6 and does not settle during controlled centrifugation, can be considered as native soluble protein. Figure S1 shows the percentage of protein than remains intact, i.e. soluble at two temperatures: 40 and 60ºC during 180 min. These temperatures correspond to the minimum and maximum studied in this investigation. Skim milk heated at 60°C and CFV 4.64 m/s lost 7.70% of the original soluble proteins, while non-significant variations were found at 40ºC, compared to the original content. Regarding the shear stress on the membrane wall τ_w, it was concluded that it would not be sufficient to produce significant alterations on the soluble proteins content, nor would there be a denaturant effect due to the passage of milk into the pump used feed the milk to the module. Since it is well known that casein micelles are highly stable even at temperatures of 70ºC, it was inferred that they did not present major alterations during the different microfiltration steps [2].

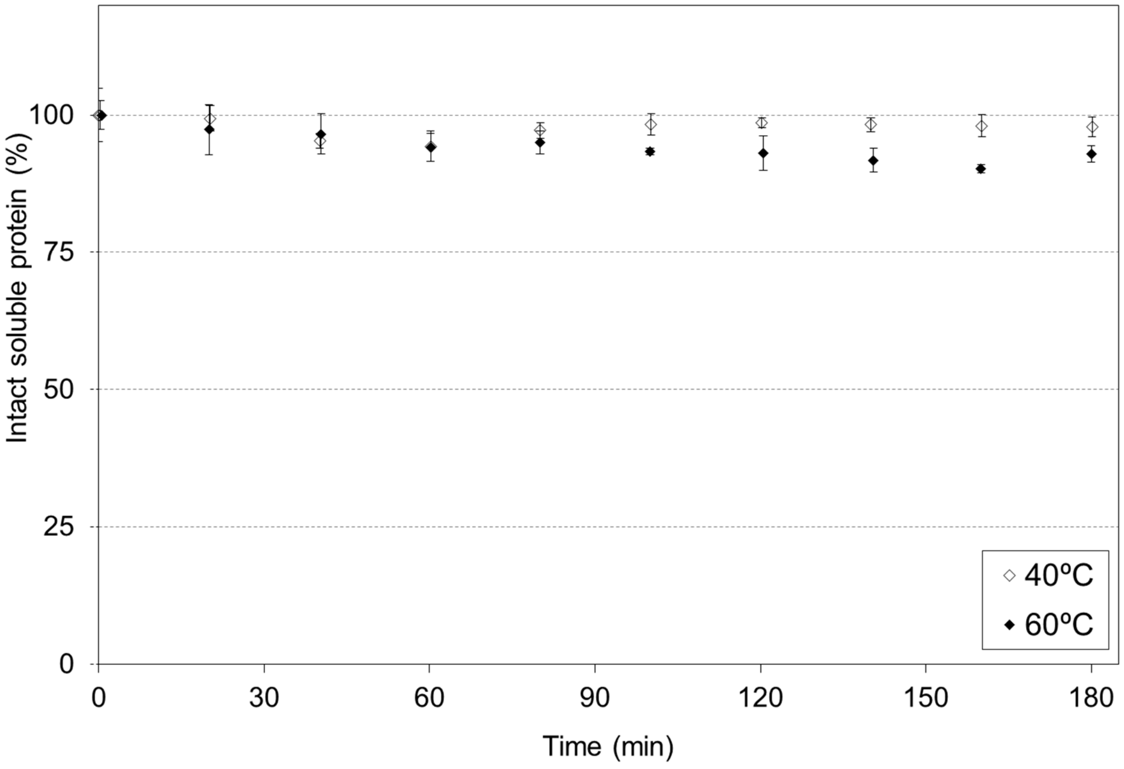

Figure S1. Evolution of the percentage of proteins respect their original concentration under extreme shear stress conditions: 231 Pa at 40ºC, and shear stress of 214 Pa at 60ºC.

References

1. Pelegrine, D.H.G.; Gasparetto, C. a. Whey proteins solubility as function of temperature and pH. *LWT - Food Sci. Technol.* **2005**, *38*, 77–80, doi:10.1016/j.lwt.2004.03.013.
2. Walstra, P. On the stability of casein micelles. *J. Dairy Sci.* **1990**, *73*, 1965–1979.
